# Saikosaponin D Inhibited IL-1β Induced ATDC 5 Chondrocytes Apoptosis *In Vitro* and Delayed Articular Cartilage Degeneration in OA Model Mice *In Vivo*

**DOI:** 10.3389/fphar.2022.845959

**Published:** 2022-03-18

**Authors:** Xinhui Wu, Kangxian Zhao, Xiaoxin Fang, Feng Lu, Pu Cheng, Xiaoting Song, Weikang Zhang, Can Yao, Jiling Zhu, Haixiao Chen

**Affiliations:** ^1^ Department of Orthopedics, Taizhou Hospital of Zhejiang Province Affiliated to Wenzhou Medical University, Linhai, China; ^2^ Wenzhou Medical University, Wenzhou, China; ^3^ Zhejiang University School of Medicine, Hangzhou, China; ^4^ Taizhou Hospital of Zhejiang Province, Zhejiang University, Linhai, China

**Keywords:** Saikosaponin D, osteoarthritis, Nrf2, HO-1, ROS, NF-κB, therapy

## Abstract

Osteoarthritis (OA) is the most common joint disease in the elderly, characterized by cartilage degradation and proliferation of subchondral bone. The pathogenesis of OA involves a variety of inflammatory mediators, including nitric oxide (NO), prostaglandin E2 (PGE2), tumor necrosis factor (TNF)-α, and interleukin (IL)-1β. From the molecular mechanism, the nuclear factor-erythroid 2-related factor (Nrf2)/heme oxygenase-1 (HO-1) pathway and the expression of ROS regulated the production of the above inflammatory mediators. Saikosaponin D (SSD), which is an active ingredient isolated from Bupleurum, has various biological functions. In this study, IL-1β was used as a pro-inflammatory factor to create an *in vitro* OA model. According to the results of high-density culture, qPCR, ROS measurement, Western blot, and immunofluorescence, SSD activated the Nrf2/HO-1/ROS axis, inhibited the production of inflammatory mediators, and protected against ECM destruction. The DMM mouse model was used as a model of OA in mice. From the results of safranin O/fast green staining, hematoxylin–eosin staining, tartrate-resistant acid phosphatase (TRAP) staining, and OARSI scores, SSD protected against the mice knee articular cartilage degeneration and reduced the number of osteoclasts in the subchondral bone. Experimental results found that SSD suppressed IL-1β–induced differentiated ATDC 5 chondrocytes apoptosis *via* the Nrf2/HO-1/ROS axis *in vitro*. SSD delayed the progression of OA in DMMs model mice *in vivo*. Therefore, SSD has the potential to become a drug for clinical treatment of OA.

## Introduction

Osteoarthritis (OA) is one of the most common chronic degenerative joint diseases, characterized by narrowing of the joint space, cartilage degradation, and proliferation of subchondral bone (Martel-Pelletier et al., 2016). The pathology of this disease is very complicated, and the specific mechanism is still controversial. A large amount of evidence indicates that pro-inflammatory factors such as tumor necrosis factor (TNF)-α and interleukin (IL)-1β play a key role in the pathogenesis of OA (Kapoor et al., 2011; Chu et al., 2014). IL-1β could induce the expression of inflammatory mediators including nitric oxide (NO) and prostaglandin E2 (PGE2) (Kapoor et al., 2011; Wojdasiewicz et al., 2014). These inflammatory mediators accumulated for a long time and destroyed the extracellular matrix (ECM), which is the main component of cartilage including collagen II and aggrecan. Eventually, the imbalance between ECM anabolism and catabolism leads to continuous chondrocyte apoptosis and cartilage degradation, which is considered to be a sign of the progression of OA (Guilak et al., 2018). Because of the lack of reliable drugs to reverse and delay OA, most patients will eventually need joint replacement surgery, leaving a huge burden on society and the economy (Cao et al., 2020; Latourte et al., 2020). Therefore, we urgently need a safe and effective drug to deal with this kind of degenerative joint disease.

Nuclear factor-erythroid 2-related factor (Nrf2) directly regulates the expression of heme oxygenase-1 (HO-1) (Saha et al., 2020). Studies demonstrated that the Nrf2/HO-1 signaling pathway could suppress the production of pro-inflammatory cytokines by immune cells. When the Nrf2 gene was knocked out, the expression of inflammatory cytokines such as COX-2, iNOS, IL-6, and TNF-α increased significantly. In addition, when the Nrf2/HO-1 axis of vascular smooth muscle cells is activated, the expression of COX2 and iNOS decreases remarkably (Ho et al., 2007). The activated Nrf2/HO-1 pathway is closely related to the inhibition of ROS and the regulated NF-κB signaling pathway (Bellezza et al., 2010; Rigoglou and Papavassiliou, 2013; Zhai et al., 2018). After IL-1β stimulation, the activation of pathways such as NF-κB and MAPKs accelerated the production of a series of inflammatory factors which caused the catabolism of chondrocytes (Sondergaard et al., 2010). The activated NF-κB pathway will cause IKBα to be degraded by the proteasome in the cytoplasm and rapidly phosphorylated. P65 enters the nucleus and specifically binds to the promoter region of downstream genes to initiate the inflammatory cascade (Sun, 2017). Therefore, the activation of the Nrf2/HO-1/ROS axis could be used as a treatment for patients with OA.

Bupleurum is a common Chinese herbal medicine with a long history and is widely used to treat inflammation and infectious diseases in Asian countries (Yuan et al., 2017). Saikosaponin D (SSD), which is an active ingredient isolated from *Bupleurum*, has anti-inflammatory, antitumor, and antiviral effects. Studies have shown that SSD can effectively inhibit late-stage autophagy and prevent EV-A71 virus infection (Li et al., 2019). Lai et al. found that SSD can target the MKK4-JNK axis to inhibit the growth of pancreatic cancer cells (Lai et al., 2020). However, the therapeutic effects of SSD on OA mice are not clear. This study aimed to investigate whether SSD treatment could suppress the IL-1β–mediated differentiated ATDC 5 chondrocytes apoptosis *in vitro*. In addition, we established a mice DMMs model to observe the protective effects of SSD treatment on articular cartilage *in vivo*.

## Materials and Methods

### Reagents and Antibodies

Saikosaponin D (purity >98%) was purchased from MCE (New Jersey, United States). DMSO (Meilunbio, Dalian, China) was used to dissolve Saikosaponin D and diluted in the cell culture until DMSO <0.1%. Recombinant mouse IL-1β was obtained from Peprotech (United States). Safranin-O and Fast Green and tartrate-resistant acid phosphatase staining (TRAP) were acquired from Solarbio (Beijing, China). Primary antibodies were applied in this study: antibodies against P65 (#8242), p-P65 (#3033), IκBα (#4814), p-IκBα (#2859), ERK (#4695), p-ERK (#4370), JNK (#9252), p-JNK (#9255), P38 (#8690), and p-P38 (#4511S) were acquired from CST (1:1,000, Cambridge, MA, United States). Anti-iNOS (1:1,000, ab178945), anti-COX2 (1:4,000, ab179800), anti-Aggrecan (1:1,000, ab3778), and anti-ADAMTS5 (1:250, ab41037) were purchased from Abcam (Cambridge, United Kingdom). Antibodies against MMP13 (1:2000, 18165-1-AP), Collagen II (1:1,000, 28459-1-AP), Nrf2 (1:2000, 66504-1-Ig), HO-1 (1:1,500, 27282-1-AP), Lamin B (1:10000, 66095-1-Ig), and Actin β (1:8,000, 66009-1-Ig) were obtained from Proteintech (Wuhan, China). Goat anti-mouse and goat anti-rabbit were acquired from Thermo Fisher (United States).

### ATDC 5 Chondrocytes Culture and Differentiation

The mice chondrocyte cell line ATDC 5 was obtained from Jennio Biotech (GuangZhou, China).

ATDC 5 chondrocytes were maintained in monolayer culture in Dulbecco’s modified Eagle’s medium (DMEM)/F12 (Hyclone; Logan, UT, United States) containing 5% fetal bovine serum (FBS; Gibco, United States) and 1% bovine serum albumin (BSA; Hyclone; United States). To induce ATDC 5 chondrocytes to the mature chondrocytes, we added insulin, transferrin, and selenium (ITS) to the culture medium. The differentiation period was 14 days, and the culture medium was changed every other day. According to previous studies, cartilage nodule formation and the elevated expression levels of type II collagen, type X collagen, and aggrecan mRNA are observed after ATDC 5 chondrocyte differentiation (Gómez et al., 2009; Morimoto et al., 2013; Abella et al., 2016).

### Cell Viability

The cytotoxicity of SSD on ATDC 5 chondrocytes was determined using a CCK-8 kit (Beyotime, Shanghai, China) according to the manufacturer’s protocol. A 6-well plate were planted with cells at a density of 8,000 cells per well and incubated with or without SSD (0, 0.5, 1, 2, 4, 8, 16, and 32 μmol/L) for 24 and 48 h. In addition, the cells were stimulated by IL-1β (10 ng/ml) or not and incubated with multiple concentrations of SSD (0, 1, 2, and 4 μmol/L) for 24 and 48 h. Each concentration of SSD was repeated 3 times. At each experimental end point, 10 μL CCK-8 kit diluted in the 100 µL DMEM/F12 was added into each well and then further incubated for 3 h at 37°C with 5% CO_2_. The absorbance of all wells was calculated using a microplate photometer at OD 450 nm (Thermo Fisher, Waltham, MA, United States).

### High-Density Culture

The mature chondrocytes at a density of 10^8^ cells/ml were seeded in a 24-well plate, 10 µl per well. After 6 h, 1 ml complete medium was added to each well and cultured for 5 days. The cells were fixed using 4% paraformaldehyde at room temperature for 15 min. After washing extensively with PBS thrice, toluidine blue o solution (solarbio, United States) was used to stain the cells. The images of the cells in a 24-well plate were observed using an EPSON V600 photo scanner (Tokyo, Japan).

### Apoptosis Detection

A 6-well plate were seeded with the cells at a density of 2 × 10^5^ cells per well. After 6 h, different concentrations of SSD (0, 2, and 4 μmol/L) were used to treat the cells under the stimulation of IL-1β (1 μmol/L) or not. An Annexin V-FITC Apoptosis Detection Kit was used to treat the cells according to the manufacturer’s instructions. The apoptosis rate of cells was quantified by flow cytometry (Moflo XDP, United States). Annexin V positive and PI negative cells were regarded as early apoptotic cells. Annexin V positive cells and PI positive cells were regarded as late apoptotic chondrocytes.

### Reactive Oxygen Species Measurement

The Reactive Oxygen Species Assay Kit (Beyotime) was used to detect the generation of cellular ROS. After special treatment of the cells in the 6-well plate (2 × 10^5^), DCFH-DA was diluted with DMEM/F12 medium (1:1,000) and added to the 6-well plate at 37°C in a cell incubator for 20 min. After washing the cells thrice with DMEM/F12 medium, the fluorescence intensity of dichlorofluorescein (DCF) fluorescence was observed using a confocal microscope (Zeiss, Japan). The cellular ROS fluorescence intensity was quantified using Image Pro Plus 6.0 software (Media Cybernetics, MD, United States).

### Quantitative Reverse Transcription Polymerase Chain Reaction Assay

A 6-well plate was planted with the cells at a density of 2 × 10^5^ cells. Various concentrations of SSD (0, 1, 2, and 4 μmol/L) were used to pretreat the cells for 30 min. The cells were then treated with or without IL-1β (10 ng/ml) and incubated with different concentrations of SSD for a day. The total RNA was extracted from the chondrocytes using TRIzol Reagent (Thermo Fisher Scientific). A cDNA synthesis kit (Cwbio; Beijing, China) was used to perform reversing transcription on the extracted RNA template to synthesize cDNA. Then ChamQ Universal SYBR qPCR Master Mix (Vazyme; Nanjing, China) was used to perform real-time quantitative PCR (qPCR) on cDNA samples according to the manufacturer’s instructions. The specific mouse sequence primers are shown in Table 1, and β-actin is used as an internal control.

**TABLE 1:** Mouse Primers for qPCR.

Gene	Forward primer	Reverse primer
β-actin	5′- AGC CAT GTA CGT AGC CAT CC-3′	5′- CTC TCA GCA GTG GTG GTG AA-3′
COX-2	5′-TCC TCA CAT CCC TGA GAA CC-3′	5′- GTC GCA CAC TCT GTT GTG CT-3′
iNOS	5′-GAC GAG ACG GAT AGG CAG AG-3′	5′-CAC ATG CAA GGA AGG GAA CT-3′
IL-6	5′-CCG GAG AGG AGA CTT CAC AG-3′	5′-TCC ACG ATT TCC CAG AGA AC-3′
TNF-α	5′-CCG GAG AGG AGA CTT CAC AG-3′	5′- GTG GGT GAG GAG CAC GTA GT- 3′

### Western Blot

The cells (density, 2 × 10^5^/well) were seeded in 6-well plates. The cells stimulated with 10 ng/ml IL-1β or not were incubated with different concentrations of SSD (0, 1, 2, and 4 μmol/L). At the end point, the cells were washed twice with ice-cold PBS and then extracted using RIPA (AMEKO, Shanghai, China) supplemented with phosphatase inhibitor and phenylmethanesulfonyl fluoride (PMSF). The concentration of total protein was normalized using a BCA protein kit (Takara, Dalian). The protein samples were separated using 12% sodium dodecylsulfate-polyacrylamide gel electrophoresis (SDS-PAGE) and subsequently transferred to 0.22-μm polyvinylidene difluoride (PVDF) membranes (Millipore, Boston, United States). After incubating with QuickBlock™ Blocking Buffer at room temperature for 30 min, the blocked PVDF membranes were incubated with the primary antibodies overnight at 4°C. On the 2nd day, tris-buffered saline with Tween 20 (TBST) was used to wash the PVDF membranes thrice and the membranes were incubated with the respective secondary antibodies for 1 h on the laboratory shaker at 37°C. Finally, the bands were acquired with an enhanced chemiluminescence reagent (Millipore, Boston, United States). ImageJ software was used to observe the density of these bands.

### Immunofluorescence Analysis

The cells were planted in 6-well plates at 2 × 10^5^ cells/well. The cells were treated with or without SSD (4 μmol/L) and stimulated with IL-1β (10 ng/ml) or not for 24 h. At the end point, the cells were then washed twice using PBS and fixed by 4% paraformaldehyde for 15 min at room temperature. The cells and nuclear membranes were permeabilized by the 0.1% Triton -100 dissolved in PBS for 5 min at room temperature and blocked with QuickBlock™ Blocking Buffer for Immunol Staining (Beyotime) for 30 min. Primary antibodies collagen (1:1,000) and P65 (1:1,000) were added to the 6-well plate and incubated with the cells overnight at 4°C. On the 2nd day, the 6-well plate was washed twice using PBS and Alexa Fluor^®^488-labeled secondary antibody or Alexa Fluor^®^594-labeled secondary antibody was added to it, and the cells were incubated at room temperature in the dark for 1 h. Finally, the nucleus was stained with DAPI for 5 min. Images of the cells were acquired by immunofluorescence microscopy (Zeiss, Japan). The fluorescence intensity was observed using Image Pro Plus 6.0 software.

### Small Interfering RNA Transfection

siRNA Nrf2 Small Interfering RNA (si-Nrf2) and siRNA negative control (si-NC) were obtained and constructed from Invitrogen (Carlsbad, United States). The special synthetic sequence of si-Nrf2 is constructed as follows: sense, 5-UUG GGA UUC ACG CAU AGG AGC ACU G3′, antisense, 5-CAG UGC UCC UAU GCG GAA UCC CAA-3′. In accordance with the protocol, lipofectamine 2000 siRNA transfection reagent (Thermo Fisher, UT, United States) was used to conduct transfection of si-NC and si-Nrf2 into the cells. The next step was to extract total cell protein for Western blot analysis.

### Construction of Binding Mode Between Saikosaponin D and Nrf2 Protein

The SSD molecular structure was constructed using ChemDraw, and we performed geometric optimization and energy minimization using Chem3D. We obtained the Nrf2 target protein crystal structure from the protein database (https://www.rcsb.org/). SSD was regarded as the small molecule ligand and Nrf2 protein target as the receptor. The center position of the Grid Box (x_center = −2.949, y_center = −3.867, z_center = 3.259) was determined based on the interaction between the small molecule and the target, and the length, width, and height are set to 60 × 60 × 60. Finally, batch molecular docking was performed using AutoDock and the results of molecular docking were analyzed. The binding effect of SSD and Nrf2 protein was visualized using Pymol2.1 software. In the calculation process, Lamarckian genetic algorithm was used for molecular docking calculation. According to the interaction between SSD and protein residues, we scored the docking of the reference compound and inferred whether the compound had a certain activity.

### A Mouse Model of Destabilization of the Medial Meniscus

Shanghai Laboratory Animal Center (SLAC) provided 24 8-week-old male C57 WT mice. To determine the effects of SSD on the mouse OA model, the mice were subjected or not to the destabilization of the medial meniscus (DMM) surgery as previously described (Ma et al., 2007; Huang et al., 2017; Jeon et al., 2020). Briefly, a 5- to 6-mm incision was made in the medial skin of the mouse right knee using a pointed blade to open the knee capsule and expose the medial meniscus. Ophthalmic scissors cut the medial collateral ligament attached to the medial tibial plateau. Finally, the joint capsule and epidermis are sutured sequentially using 6–0 sutures. According to the IACUC protocol, analgesia and antibiotics treatments were administered for 3 days after DMM surgery. The Institutional Animal Ethics Committee of Taizhou Hospital approved all animal experiments (ethic code: tzy-2021170). The Health Guide for Care and Use of Laboratory Animals was regarded as the guideline for the care and use of our experimental animals. There were four groups to which we randomly assigned mice (*n* = 6): sham group, OA group (intra-articular injection with PBS), OA treated with low-dose SSD group (intra-articular injection with 2 mg/kg SSD), and OA treated with high-dose SSD group (intra-articular injection with 4 mg/kg SSD). The dosage of SSD is determined by referring to the relevant literature (Li et al., 2020; Qin et al., 2020). Pentobarbital (40 mg/kg) by intraperitoneal injection was used to anesthetize mice, and the DMM surgery was conducted on the right knee of each mouse except the sham group. After 1 week of the DMM surgery, PBS or SSD dissolved in PBS was intraperitoneally injected every other day during the 8-week period. The mice were allowed to get food, water, and free movements in the same way after surgery. All animals were maintained at a 12-h light/dark cycle, a relative humidity of 50 ± 10% within a constant temperature of 20 ± 2°C. The mice were euthanized at the end point, and all right knee joints were fixed by 4% PFA paraformaldehyde for further analysis.

### Knee Joints Tissue Section Analysis

All knee joint specimens were decalcified in 10% ethylenediaminetetraacetic acid (EDTA) for 2 weeks. The decalcified samples were embedded in paraffin and cut into 4-μm sections. We used Safranin-O (S-O), hematoxylin and eosin (HE), and tartrate-resistant acid phosphatase staining (TRAP) staining reagents to stain the sections as previously described (Kim et al., 2014; Zhu et al., 2019). Finally, the Osteoarthritis Research Society International (OARSI) scoring system was used to evaluate the degree of destruction of articular cartilage (Glasson et al., 2010). Image ProPlus 6.0 software (MD, United States) was used to measure the number of TRAP-positive cells in the subchondral bone.

### Statistical Analysis

All experimental data were presented as mean ± S.D. The experiments *in vitro* were performed at least three times. Each group of animal experiments was randomly assigned to 6 mice. SPSS statistical software version 20.0 (IBM Corp, Armonk, NY, United States) was used for statistical analysis. Two sets of data were compared using Student’s *t*-test or the Kruskal–Wallis test. The significance between various groups was examined with one-way analysis of variance (ANOVA) with Tukey’s post hoc test. *p* values ˂ 0.05 were considered statistically significant.

## Results

### Effects of Saikosaponin D and IL-1β on ATDC 5 Chondrocytes Viability

**FIGURE 1:**
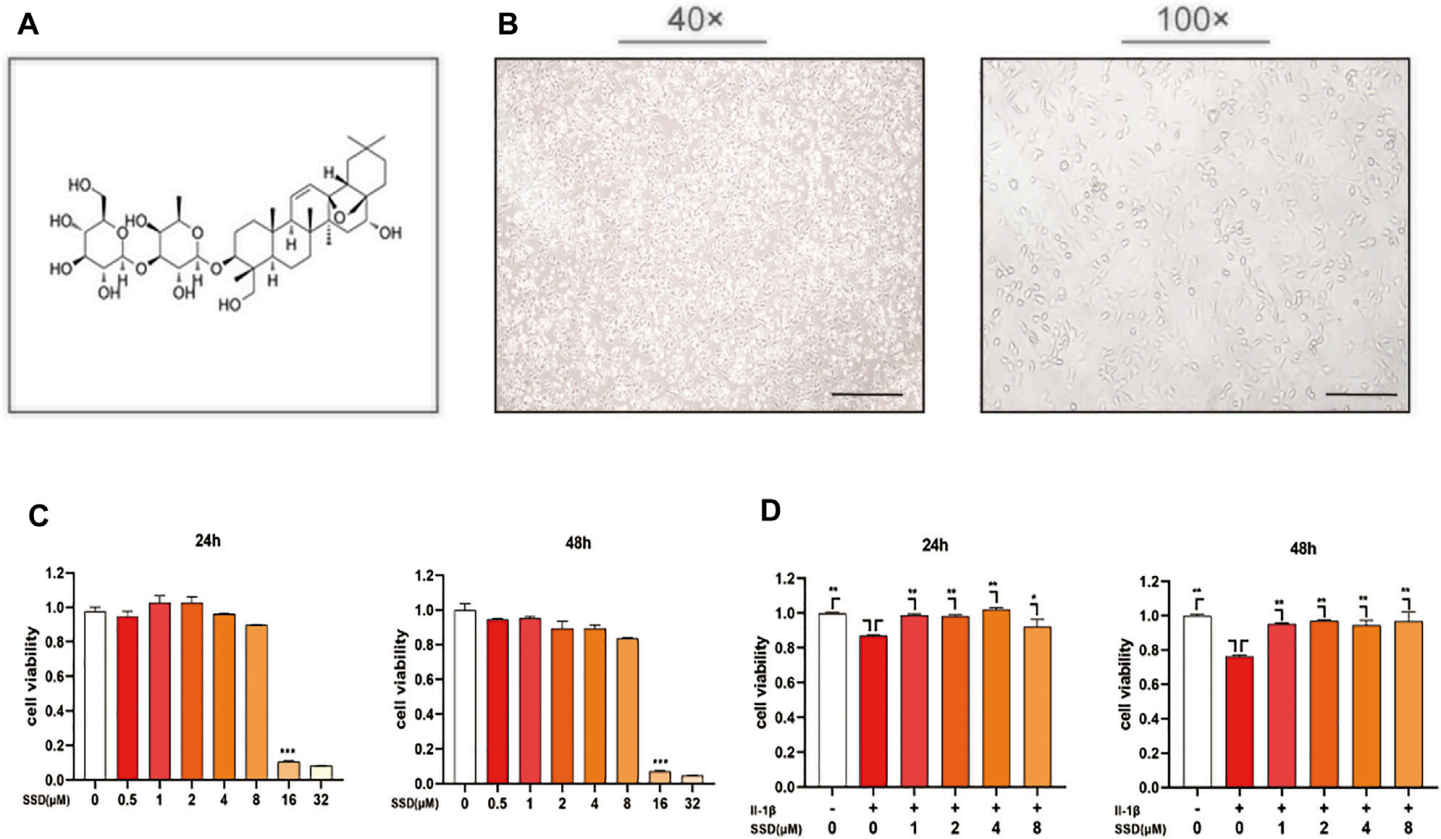
SSD improved the cell viability of the cells stimulated by IL-1β and prevented cell death. **(A)** The chemical structure of SSD. **(B)** The morphology of ATDC 5 chondrocytes under the microscope (40×; 100×; Scale, 200 μm). **(C)** Effects of various concentrations of SSD on the cell viability. **(D)** The protective effects of SSD on the cells stimulated by IL-1β.

The chemical structure of SSD is shown in Figure 1A. The morphology of ATDC 5 chondrocytes under the microscope is shown in Figure 1B. In order to determine the potential cytotoxicity of SSD, the ATDC 5 chondrocytes were incubated with or without SSD at various concentrations (0.5, 1, 2, 4, 8, 16, and 32 μmol/L) for 24 and 48 h. The cellular viability was determined using a CCK-8 assay kit. Figure 1C shows that SSD show no apparent cytotoxicity to the cells at concentrations of ≤4 μmol/L after a day or 2 days. Therefore, the concentrations of SSD (1, 2, or 4 μmol/L) were used in the following experiments. The cell viability of ATDC 5 chondrocytes decreased significantly under the stimulation of IL-1β (10 ng/ml), and the treatment of SSD reversed this phenomenon**.** Compared with IL-1β stimulation alone, ATDC 5 chondrocytes had a better viability after SSD treatment (Figure 1D).

### Saikosaponin D Attenuated IL-1β–Induced Differentiated ATDC 5 Chondrocyte Apoptosis

**FIGURE 2:**
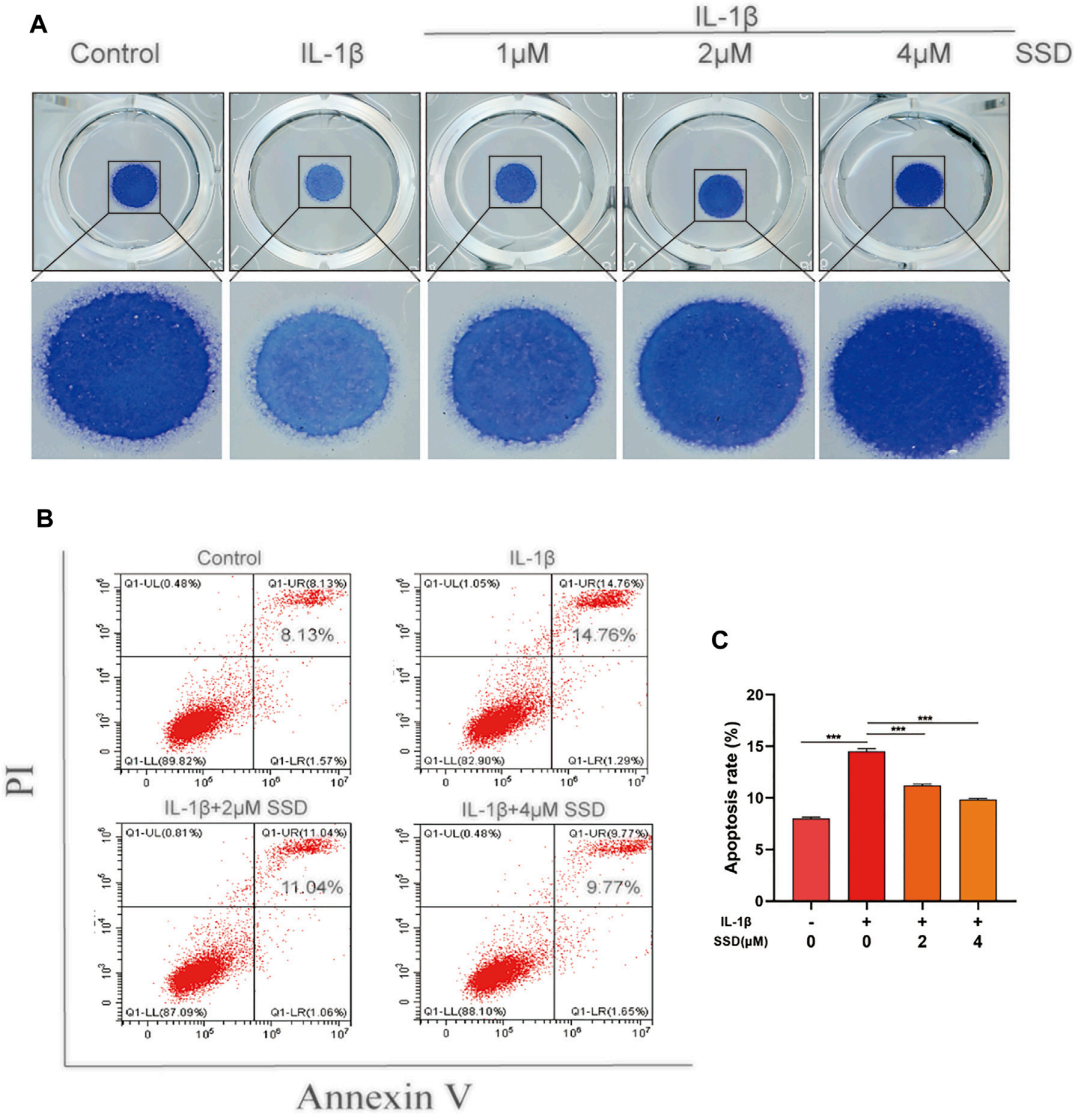
SSD prevented IL-1β–induced differentiated ATDC 5 chondrocyte apoptosis. **(A)** The cells (density, 10^8^/ml) stimulated by IL-1β were seeded in 24-well plates and treated with or without different concentrations of SSD for 5 days. Images of the cells stained with toluidine blue o solution. **(B)** The cellular apoptotic ratios in different groups were quantified by flow cytometry. **(C)** Statistical analysis of the cellular apoptosis results between groups. Mean ± SD was applied with all data. The experiments *in vitro* were performed independently at least 3 times. ***p < 0.001, **p < 0.01, *p < 0.05.

As shown in Figure 2A, the protective effects of SSD (1, 2, and 4 μmol/L) on the mature chondrocytes stimulated by IL-1β were detected. The results of high-density culture showed that the cells’ density increased significantly and the area of toluidine blue o solution staining was larger than the cells treated with IL-1β alone after SSD treatment. According to the results of flow cytometry (Figures 2B,C), the cell apoptosis under IL-1β stimulation was inhibited by SSD in a dose-dependent manner. Especially when the SSD concentration was 4 μmol/L, the cellular apoptosis rate was significantly lower than that of the IL-1β group.

### The Inhibition of Saikosaponin D on the Secretion of Inflammatory Factors by the Mature Chondrocytes

**FIGURE 3:**
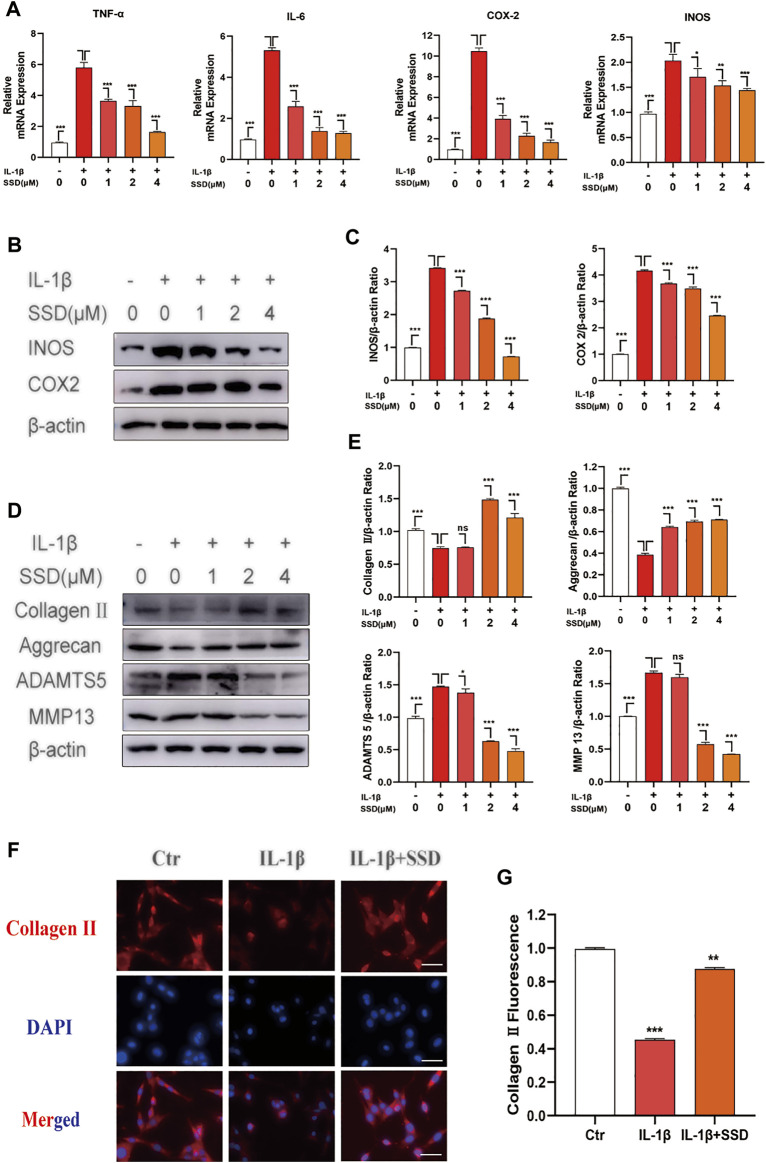
SSD attenuated the production of inflammation-related factors and protected the ECM from damage under the stimulation of IL-1β. The mature chondrocytes stimulated by 10 ng/ml IL-1β or not were incubated with different concentrations of SSD (0, 1, 2, and 4 μM) for 2 days. The extracted RNA and total protein were used for further analysis by Western blot and qPCR. **(A)** SSD inhibited the expression level of inflammatory cytokine related marker genes in a dose-dependent manner. **(B)** SSD suppressed the production of INOS and COX2, especially at 4 μM. **(C)** The quantification of INOS and COX2 protein expression levels. **(D)** SSD reduced the expression level of ADAMTS 5 and MMP13 and increased the expression level of Aggrecan and Collagen II in a dose-dependent manner. **(E)** The quantification of ADAMTS 5, MMP13, Aggrecan, and Collagen II protein expression levels. **(F)** Immunofluorescence was conducted to visualize Collagen II. **(G)** The quantification of Collagen II fluorescence intensity (original magnification, ×200; scale, 15 μm). Mean ± SD was applied with all data. The experiments *in vitro* were performed independently at least 3 times. ***p < 0.001, **p < 0.01, *p < 0.05.

The cells were treated with different concentrations of SSD (0, 1, 2, and 4 μmol/L) under IL-1β–induced inflammatory conditions. Related inflammatory factors were detected by Western blot and qPCR. According to our experimental results, IL-1β upregulated the expression level of inflammation-related maker genes: TNF-α, IL-6, COX2, and INOS, whereas high concentration of SSD (4 mol/L) could decrease the degree to baseline (Figure 3A). Similarly, Figures 3B,C shows that SSD significantly decreased the protein level of COX2 and INOS in a dose-dependent manner. Together, these data found that SSD could reverse the upregulation trend of inflammatory cytokines under IL-1β stimulation at the gene and protein levels.

### Saikosaponin D Inhibited IL-1β–Induced Production of Extracellular Matrix Catabolic

To evaluate the protective effects of SSD on IL-1β–induced degradation of the ECM, the ECM components of chondrocytes were determined by using Western blot analysis and immunofluorescence. As shown in Figures 3D,E, IL-1β stimulation remarkably accelerated the production of ADAMTS 5 and MMP13 compared with the negative control group, while this trend was reversed by SSD pretreatment. Meanwhile, the expression levels of type II collagen and aggrecan decreased significantly after stimulation with IL-1β (10 ng/ml) for 24 h. SSD treatment protected these two kinds of ECM compositions from damage. In addition, the immunofluorescence results showed that the fluorescence intensity of type II collagen significantly decreased when the cells were stimulated with IL-1β alone, but the fluorescence intensity was increased after SSD treatment (Figures 3F,G). Therefore, SSD could promote ECM anabolism and suppress ECM catabolism.

### The Binding Mode Between Saikosaponin D and Nrf2 Protein

**FIGURE 4:**
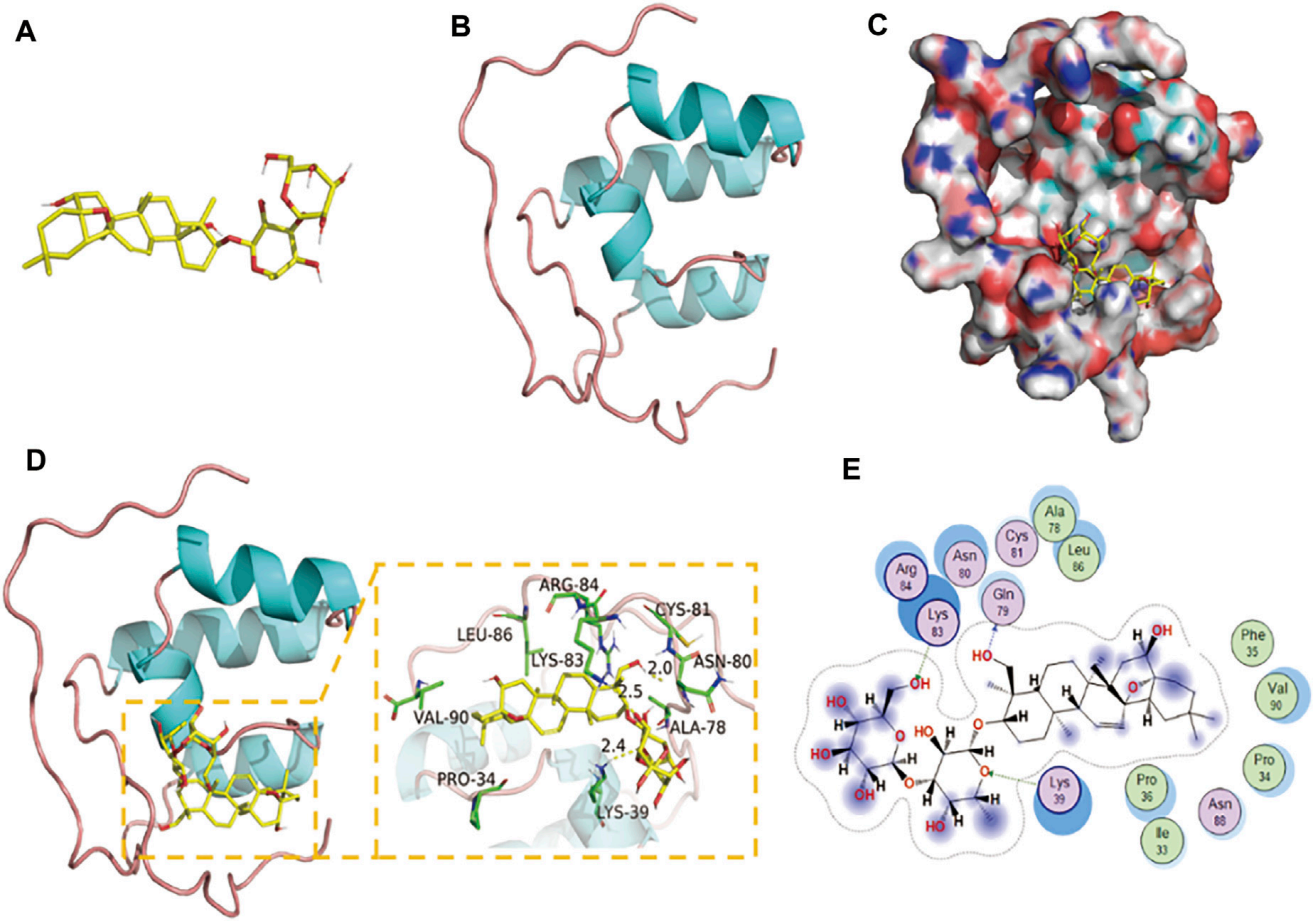
Binding mode of Nrf2 with SSD. **(A)** The 3D structure of SSD. **(B)** The 3D structure of Nrf2. **(C)** The surface of Nrf2 with SSD. **(D,E)** The detail binding mode of Nrf2 with SSD. The backbone of protein was rendered in tube and colored in blue. SSD are rendered in yellow. Yellow dash represents hydrogen bond distance.

According to the results of molecular docking, SSD has a strong binding effect with NrF2 target protein (binding energy of −8.19 kcal/mol) (Figures 4A–C). From the binding mode of SSD and Nrf2 protein, the amino acid residues that SSD binds to the active site of Nrf2 protein include LEU-86, ARG-84, LYS-83, CYS-81, ASN-80, ALA-78, LYS-39, PRO-34, VAL-90, etc. (Figure 4D). One end of the SSD is very hydrophobic. Multiple hydrophobic amino acids (LEU-86, ALA-78, PRO-34, and VAL-90) of the target protein form a strong hydrophobic interaction with the SSD, which helps stabilize the small molecules in the protein pocket. The other end of the SSD has more active groups, which can form strong hydrogen bond interactions with LYS-83, LYS-39, and GLN-79 (Figure 4E). The hydrogen bond distances are 2.5, 2.4, and 2.0 Å, respectively. In summary, the SSD and Nrf2 protein are well matched and strongly bound, and the complex formed is relatively stable.

### The Activation of Saikosaponin D on the Nrf2/HO-1/Reactive Oxygen Species Axis

**FIGURE 5:**
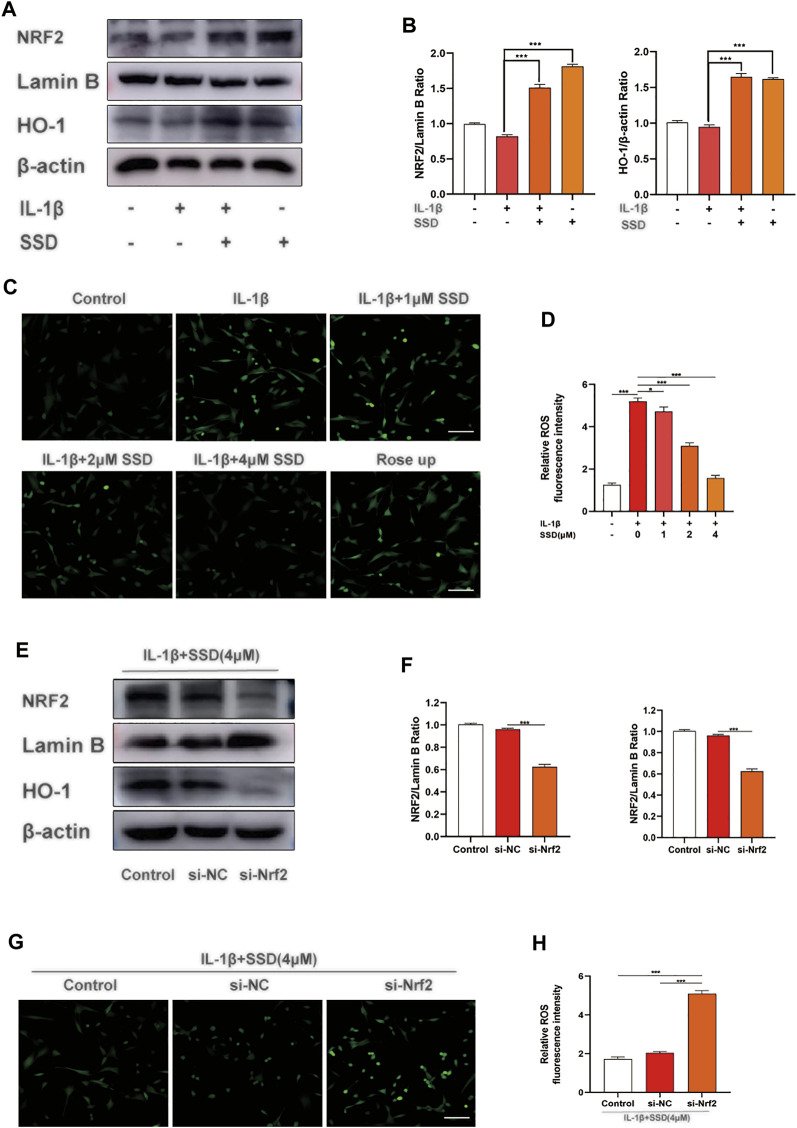
SSD activated the Nrf2/HO-1 pathway and suppressed the generation of ROS. **(A)** SSD increased the expression levels of Nrf2 and HO-1regardless of the existence of IL-1β stimulation. **(B)** The quantification of Nrf2 and HO-1 protein expression levels. **(C)** Immunofluorescence was conducted to visualize ROS level. SSD decreased cellular ROS immunofluorescence intensity under IL-1β stimulation. **(D)** The quantification of ROS immunofluorescence intensity. **(E)** SSD could not increase the expression level of HO-1 after Nrf2 was knocked out. **(F)** The quantification of Nrf2 and HO-1 protein expression levels. **(G)** SSD has no effects on cellular ROS level under IL-1β stimulation after Nrf2 was knocked out. **(H)** The quantification of immunofluorescence intensity (original magnification, ×200; scale, 15 μm). Mean ± SD was applied with all data. The experiments *in vitro* were performed independently at least 3 times. ***p < 0.001, **p < 0.01, *p < 0.05.

According to previous studies, Nrf2 is maintained in the cytoplasm by Keap1 (Lu et al., 2016; Yamamoto et al., 2018). When Nrf2 is activated, Nrf2 is phosphorylated and translocated to the nucleus and quickly accelerates the expression of downstream HO-1 (Loboda et al., 2016). The activated Nrf2/HO-1 axis suppresses the production of ROS and the NF-κB signaling pathway in rheumatoid arthritis fibroblast like-synoviocytes (RA-FLSs) (Zhai et al., 2018). From the results in Figures 5A,B, SSD treatment significantly increased the expression of Nrf2 and HO-1 in a dose-dependent manner regardless of whether the cells were stimulated with IL-1β. Similarly, the results of ROS detection showed that SSD significantly reduced the fluorescence intensity of cellular ROS compared to the IL-1β group (Figures 5C,D). The results showed that SSD could activate the Nrf2/HO-1 axis and reduce the generation of ROS.

### Nrf2 siRNA Suppressed the Activation of the Nrf2/HO-1 Pathway and the Generation of Reactive Oxygen Species

According to the results of Western blot, Nrf2 knockdown significantly reduced the expression level of HO-1. Compared with the si-NC group, SSD could not activate the Nrf2/HO-1 signal pathway after Nrf2 was knocked down (Figures 5E,F). In addition, the results of ROS detection indicated that the production of ROS was significantly increased in the si-NC group (Figures 5G,H). At this time, SSD could not play the role of activating the Nrf2/HO-1/ROS axis.

### Effects of Saikosaponin D on NF-κB and Mapks Signaling Pathways in Differentiated ATDC 5 Chondrocytes

**FIGURE 6:**
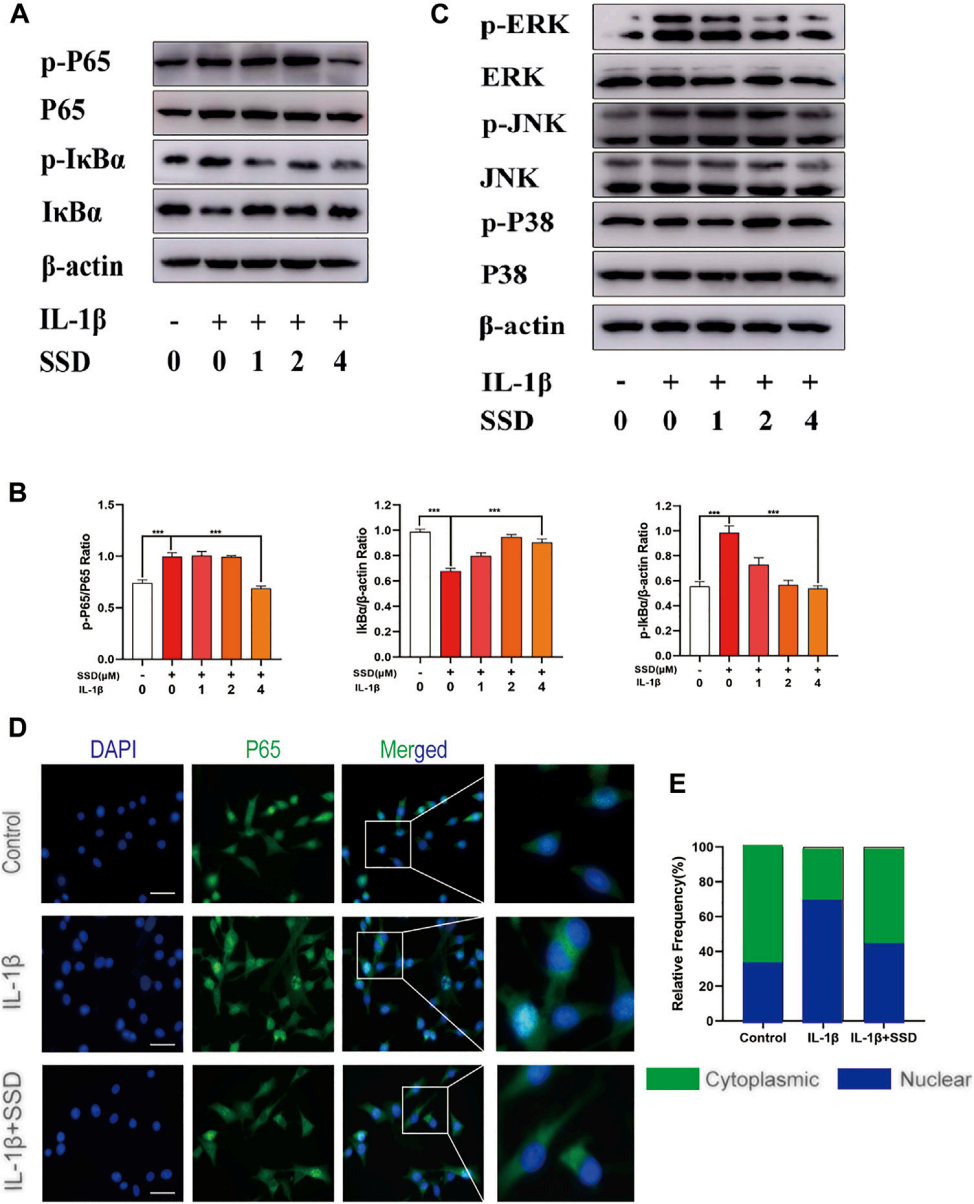
Inhibition of SSD on the NF-κB signaling pathway. **(A)** SSD prevented IκBα from being degraded and inhibited the phosphorylation level of p-P65 and p-IκBα. **(B)** Above protein expression level was quantified using ImageJ. **(C)** SSD had no effects on Mapks signaling pathway–related proteins including P38, p-P38, ERK, p-ERK, JNK, and p-JNK. **(D)** Immunofluorescence was conducted to detect the P65 nuclear translocation. **(E)** The quantification of P65 fluorescence intensity (original magnification, ×200; scale, 15 μm). Mean ± SD was applied with all data. The experiments *in vitro* were performed independently at least 3 times. ***p < 0.001, **p < 0.01, *p < 0.05.

To further investigate the molecular mechanism of SSD on the NF-κB and Mapks signaling pathways in differentiated ATDC 5 chondrocytes, related protein expression levels in these signaling pathways were measured by Western blot analysis. Figures 6A,B showed that IκBα was degraded after IL-1β stimulation compared with the negative control group. The phosphorylation of p65 and IκBα was remarkably upregulated in the IL-1β group. However, the phosphorylation levels of P65 and IκBα was decreased significantly by SSD treatment. The Mapks play a key regulatory role in the production of pro-inflammatory factors. We observed that IL-1β enhanced the phosphorylation level of ERK, JNK, and P38, but SSD could not significantly reduce related phosphorylation levels (Figure 6C). Consistent with the results of immunofluorescence, P65 had a higher fluorescence intensity in the nucleus in the IL-1β group and SSD treatment reduced the fluorescence intensity (Figures 6D,E). The results indicated that SSD prevented the P65 nuclear translocation mediated by the IκBα pathway.

### Saikosaponin D Attenuated the Progression of Cartilage Degeneration in Osteoarthritis Model Mice

**FIGURE 7:**
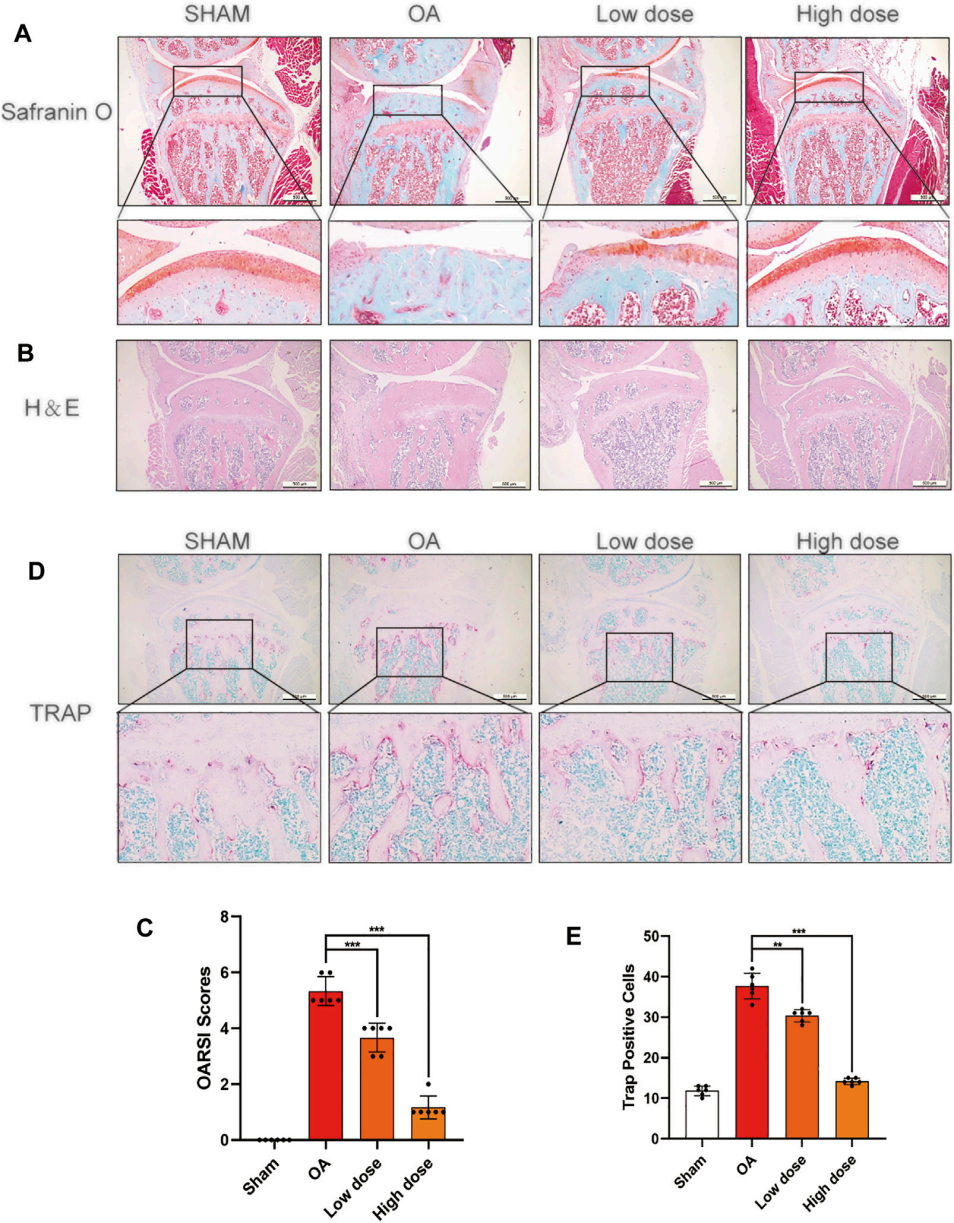
SSD treatment delayed the progression of cartilage destruction in OA mice and decreased the osteoclasts in the knee joints’ subchondral bone. **(A,B)** The S-O staining and H&E staining images of the right knee articular cartilage of 8-week-old male mice. SSD protected the mouse’s right knee articular cartilage from damage. **(C)** The knee joint damage of each group was evaluated according to Osteoarthritis Research Society International (OARSI) scores. **(D)** The images of subchondral bone TRAP staining in male mice at 8 weeks. The activity of osteoclasts was weakened in the subchondral bone of mice right joints after SSD treatment. **(E)** The TRAP-positive cells were measured in the subchondral bone of each group using ImageJ. Mean ± SD was applied with all data. There were 6 randomly assigned male mice in each group. ***p < 0.001, **p < 0.01, *p < 0.05.

Based on the results *in vitro*, an *in vivo* OA model mouse was established to further investigate whether SSD could delay the progression of OA. OARSI score, H&E staining, S-O staining, and TRAP staining were used to perform histological analysis on mice of each group. The ratio of the red-stained area to the total area (red/total area) in the OA histone proteoglycan S-O staining was reduced in the OA group, indicating that the associated cartilage matrix was degraded. However, the red area ratio increased significantly in SSD intraperitoneal injection groups (low- and high-dose groups) (Figure 7A). For the H&E staining results, the number of chondrocytes decreased remarkably and the thickness and shape of the articular cartilage was irregular in the OA group. The shape of the articular cartilage was more regular and there were more chondrocytes in SSD low- and high-dose groups (Figure 7B). According to the results of TRAP staining of histological sections, the number of TRAP-positive cells were decreased in SSD low- and high-dose groups compared with the OA group (Figures 7D,E). Consistent with the above results, the OARSI scores of SSD treatment groups were significantly lower than that of the OA group and closer to that of the sham group (Figure 7C). Therefore, SSD intraperitoneal injection suppressed the progression of cartilage degeneration in OA model mice.

## Discussion

Osteoarthritis (OA) is a common joint degenerative disease in the elderly. Both systemic and local factors are involved in the development of the disease (Martel-Pelletier et al., 2016). The clinical features of OA mainly include pain, joint dysfunction, and destruction of articular cartilage, resulting in restricted daily activities and even physical disability (Bijlsma et al., 2011). IL-1β and TNF-α are key pro-inflammatory cytokines involved in the pathophysiology of OA (Hosseinzadeh et al., 2016). Inhibiting the production of IL-1β and TNF-α could protect articular cartilage and reduce knee joint degeneration (Kapoor et al., 2011). However, the main purpose of current OA treatment is to relieve pain and improve joint function, including NSAIDs, analgesics, and joint lubricants (Kapoor et al., 2011). More and more studies have shown that plant and fruit extracts can be used in the treatment of related pro-inflammatory factor–mediated diseases (Azab et al., 2016). A recent study found that tangeretin downregulates IL-1β–induced secretion of inflammatory mediators and delays OA progression in mice (Shi et al., 2022). In our study, SSD is a triterpene saponin compound extracted from Bupleurum, which has various pharmacological activities such as anti-inflammatory, anti-oxidant, and antitumor activities (Li et al., 2019; Lai et al., 2020). This study found that SSD inhibited the expression level of inflammatory factors in the mature chondrocytes stimulated with IL-1β and increased the synthesis of the ECM *in vitro*.

Studies have found that IL-1β is an important inflammatory cytokine, which is significantly elevated in the knee synovial fluid of OA patients, and is closely related to the occurrence and development of OA. Therefore, the *in vitro* model of OA could be simulated by IL-1β–induced chondrocyte damage. The mature chondrocytes induced by IL-1β have an inflammatory response, and the expression of iNOS and COX2 increased at the mRNA and protein levels (Huang et al., 2009; Wojdasiewicz et al., 2014). Among them, iNOS belongs to the family of nitric oxide synthase (NOS) enzymes and is involved in NO synthesis (Cinelli et al., 2020). COX-2 can produce PGE2, an important inflammatory factor in the development of OA (Tong et al., 2018). Overexpression of inflammatory factors such as PGE2 and NO can disrupt the balance between ECM anabolism and catabolism (Amin et al., 2000). Studies have shown that inhibition of PGE2 and NO has a positive effect on the protection of articular cartilage (Wojdasiewicz et al., 2014). In addition, NO and PGE2 can induce chondrocytes to synthesize and release matrix metalloproteinases (MMPs) after reaching a certain amount of accumulation. MMPs are a major member of the proteolytic enzyme family, which participate in the process of chondrocyte apoptosis and destroy ECM components (aggrecan and collagen II). According to the results of previous studies, with the progress of OA, the levels of MMP-3 and MMP-13 increased significantly (Mehana et al., 2019). ADAMTS5 belongs to the ADAMTS protein family and participates in cartilage degradation during the OA progression. Studies have shown that ADAMTS5 cleaves proteoglycans in the early stages of OA. The loss of aggrecan in human OA cartilage explants is significantly reduced after transfection with siRNA, indicating that it may be a potential therapeutic target for OA (Hoshi et al., 2017; Malemud, 2019). When the ECM is destroyed, chondrocytes exposed to non-physiological load undergo apoptosis. At the same time, the high concentration of proteolytic enzymes accumulated by apoptosis will degrade aggrecan and collagen II. These factors lead to the further development of OA.

The NF-κB pathway involved in the progression of OA disease was regulated by the Nrf2/HO-1 axis (Bellezza et al., 2010). In the resting state, NF-κB binds to IκBα and is located in the cytoplasm. When stimulated by IL-1β, IκBα degraded and released P65 into the nucleus. The expression level of related inflammatory genes was accelerated, including COX-2, iNOS, MMPs, and ADAMTS (Rigoglou and Papavassiliou, 2013). As mentioned earlier, these inflammatory factors destroyed the balance between anabolism and catabolism of the ECM. Our experimental results showed that SSD activated the Nrf2/HO-1/ROS pathway and suppressed the IL-1β–induced NF-κB signaling pathway activation in the mature chondrocytes. Therefore, SSD attenuated IL-1β–induced chondrocyte apoptosis *via* the Nrf2/HO-1/ROS axis.

We performed mice DMM surgery on an *in vivo* research model of OA (Jia et al., 2019; Yan et al., 2020). Compared with the sham group, mice undergoing DMM surgery experienced joint space stenosis, cartilage erosion, and a decrease in the number of chondrocytes. Compared with the sham group, there were larger cartilage matrixes, more chondrocytes, fewer osteoclasts, and lower OARSCI scores in SSD intraperitoneal injection groups. Our experimental results showed that SSD could suppress cartilage destruction and chondrocyte loss *in vivo* OA model mice.

In summary, this study demonstrated that SSD inhibited differentiated ATDC 5 chondrocytes inflammatory apoptosis *via* the Nrf2/HO-1/ROS axis *in vitro* and suppressed cartilage destruction and chondrocyte loss *in vivo*. All above findings showed that SSD might have the potential to become a therapeutic drug for human OA in the future.

## Data Availability

The original contributions presented in the study are included in the article/Supplementary Materials, further inquiries can be directed to the corresponding author.
